# One year follow-up after a randomized controlled trial of a 130 g/day low-carbohydrate diet in patients with type 2 diabetes mellitus and poor glycemic control

**DOI:** 10.1371/journal.pone.0188892

**Published:** 2017-12-04

**Authors:** Junko Sato, Akio Kanazawa, Chie Hatae, Sumiko Makita, Koji Komiya, Tomoaki Shimizu, Fuki Ikeda, Yoshifumi Tamura, Takeshi Ogihara, Tomoya Mita, Hiromasa Goto, Toyoyoshi Uchida, Takeshi Miyatsuka, Chie Ohmura, Takehito Watanabe, Kiyoe Kobayashi, Yoshiko Miura, Manami Iwaoka, Nao Hirashima, Hirotaka Watada

**Affiliations:** 1 Department of Metabolism & Endocrinology, Juntendo University Graduate School of Medicine, Bunkyo-ku, Tokyo, Japan; 2 Department of Diabetes, Endocrinology and Metabolism, Juntendo University Shizuoka Hospital, Izunokuni-shi, Shizuoka, Japan; 3 Center for Therapeutic Innovations in Diabetes, Juntendo University Graduate School of Medicine, Bunkyo-ku, Tokyo, Japan; 4 Department of Nutrition, Juntendo University Hospital, Bunkyo-ku, Tokyo, Japan; 5 Sportology Center, Juntendo University Graduate School of Medicine, Bunkyo-ku, Tokyo, Japan; 6 Center for Identification of Diabetic Therapeutic Targets, Juntendo University Graduate School of Medicine, Bunkyo-ku, Tokyo, Japan; Weill Cornell Medical College Qatar, QATAR

## Abstract

**Background & aims:**

Recently, we conducted a prospective randomized controlled trial (RCT) showing that a 6-month 130g/day low-carbohydrate diet (LCD) reduced HbA1c and BMI more than a calorie restricted diet (CRD). [1] To assess whether the benefits of the LCD persisted after the intensive intervention, we compared HbA1c and BMI between the LCD and CRD groups at 1 year after the end of the 6-month RCT.

**Methods:**

Following the end of the 6-month RCT, patients were allowed to manage their own diets with periodic outpatient visits. One year later, we analyzed clinical and nutrition data.

**Results:**

Of the 66 participants in the original study, 27 in the CRD group and 22 in the LCD group completed this trial. One year after the end of the original RCT, the carbohydrate intake was comparable between the groups (215 [189–243]/day in the CRD group and 214 (176–262) g/day in the LCD group). Compared with the baseline data, HbA1c and BMI were decreased in both groups (CRD: HbA1c -0.4 [-0.9 to 0.3] % and BMI -0.63 [-1.20 to 0.18] kg/m^2^; LCD: HbA1c -0.35 [-1.0 to 0.35] % and BMI -0.77 [-1.15 to -0.12] kg/m^2^). There were no significant differences in HbA1c and BMI between the groups.

**Conclusions:**

One year after the diet therapy intervention, the beneficial effect of the LCD on reduction of HbA1c and BMI did not persist in comparison with CRD. However, combining the data of both groups, significant improvements in HbA1c and BMI from baseline were observed. Although the superiority of the LCD disappeared 1 year after the intensive intervention, these data suggest that well-constructed nutrition therapy programs, both CRD and LCD, were equally effective in improving HbA1c for at least 1 year.

**Trial registration:**

University Hospital Medical Information Network (UMIN) ID000010663

## Introduction

Diet is one of the basic therapies for patients with type 2 diabetes mellitus (T2DM). Both calorie-restricted diets (CRDs) and low-carbohydrate diets (LCDs) are regarded as useful therapies for patients in Western countries. [2–11] However, differences in food cultures among countries markedly affect the efficacy of diet therapies, thus it is important to evaluate the methodology of each diet therapy in individual countries. Regarding Japanese patients, except for 1 small randomized controlled trial (RCT), [12] no previous RCTs have compared the effects of CRDs and LCDs. Thus, we recently conducted a 6-month RCT to compare the outcome of a LCD limiting carbohydrate intake to 130 g/day, with that of a CRD, in obese Japanese patients with uncontrolled T2DM. [1] We found that HbA1c decreased more in the LCD group (-0.65 [-1.53 to -0.10] %) than in the CRD group ((0.00 [-0.68 to 0.40] %), p<0.01). Also, the decrease in body mass index (BMI) in the LCD group (-0.58 [-1.51 to -0.16] kg/m^2^) exceeded that observed in the CRD group (p = 0.03). Thus, we concluded that a 6-month LCD is a potentially useful nutrition therapy for Japanese patients who cannot adhere to a CRD. [1]

Long-term treatment adherence is important to prevent or reduce the progression of diabetic complications. Evaluation of treatment continuity will provide useful information for clinical practice. Thus, in this study, we assessed whether the significant improvement of HbA1c and BMI observed in our previous study persisted at 1 year after the intensive intervention. This study is the extension of the previously performed RCT. [1]

## Materials and methods

### Participants

In the RCT, we recruited patients with T2DM from the Outpatient Clinic of Juntendo University Hospital. The following inclusion criteria were applied at study registration: 1) age >20 but <75 years; 2) HbA1c (NGSP) >7.5% for more than 3 months, with HbA1c fluctuations within ±0.5%; 3) BMI >23 kg/m^2^; and 4) previous participation in least 2 educational programs on CRDs conducted by dieticians. The exclusion criteria were as follows: 1) proliferative retinopathy, 2) severe neuropathy, 3) serious kidney disease (serum creatinine level >2.0 mg/dL and/or microalbuminuria), 4) serious liver disease excluding fatty liver (aspartate aminotransferase and/or alanine aminotransferase levels >100 IU/L), 5) acute heart failure within 3 months or apparent chronic heart failure, 6) active malignancy, 7) serious pancreatic disease, 8) pregnancy, 9) serious infectious disease, 10) traumatic injury, 11) alcohol dependency, 12) not suitable for the study, and 13)patients who eat carbohydrate less than 130g/day before registration.

The study protocol including the extension after the RCT was approved by the Human Ethics Committee of Juntendo University (approval number 12–200) and registered with University Hospital Medical Information Network[UMIN: https://upload.umin.ac.jp/cgi-open-bin/ctr/ctr.cgi?function=brows&action=brows&recptno=R000012471&type=summary&language=J] (ID 000010663). Written informed consent was obtained from each patient before study enrollment.

### Study design

The open-label, 2-arm, randomized controlled study was performed at the outpatient clinic of Juntendo University Hospital from September 2013 to November 2014 (Recruitment period: from September 2013 to May 2014). Study team doctors enrolled participants.

According to the previous study including 24 Japanese patients, [12] HbA1c from the baseline after the intervention of LCD decreased 0.6% and of CRD decreased 0.2%. From this study, the difference in HbA1c reduction between two groups was estimated as 0.4% with 0.5% of standard deviation (SD). With the two-sided α level of 5% and a power (1-β) of 90%, the appropriate number of study subject was considered to be sixty-six patients (33 patients for each group). Sixty-six patients who met the above criteria were assigned randomly to either a CRD or LCD for 6 months. [1] Randomization was achieved by the minimization and biased coin method. The minimization variables are HbA1c and BMI of patients. Allocation sequence conducted by third party organization (Soiken, Inc., Osaka, Japan) was concealed until interventions were assigned.

After the end of the 6-month RCT, the patients were allowed to manage their own diets and made periodic visits to the outpatient clinic of Juntendo University Hospital. We obtained their clinical and nutrition data 1 year after the end of the RCT. The follow-up period was from February 2015 to November 2015. The primary endpoint was the change in HbA1c level from baseline to 1 year after the end of the 6-month RCT. The secondary endpoints were changes in BMI (Body weight), frequency of hypoglycemia and lipid metabolism from baseline to 1 year after the end of the 6-month RCT.

### Intervention

The study spanned 18 months, including the initial 6-month RCT period. In the RCT, the patients were followed up regarding the intervention by the same physicians at an outpatient clinic, and by dieticians at 0, 1, 2, 4, and 6 months. After the end of the RCT, the patients discontinued the intervention and followed up with their physicians routinely almost every 2 months.

Nutrition data were collected by dieticians with 3-day weighed/measured food records at 0, 1, 2, 4, 6 and 18 months. The calorie intake and nutritional breakdown were calculated using the analysis software Super Nutrition Calculation System, Healthy Maker Pro 501 Series (Mushroomsoft, Okayama, Japan), which is generally used by Nutrition Departments at Japanese hospitals.

At all visits, body weight was measured by the staff. Blood samples were obtained after overnight fasts. Serum lipids (total cholesterol [T-CHO], High density lipoprotein cholesterol [HDL-C], Low density lipoprotein cholesterol [LDL-C], triglycerides), fasting blood glucose (FBG), HbA1c, liver enzymes, and serum creatinine were measured with standard techniques at 0, 1, 2, 4, 6, 12, and 18 months.

Each patient completed the Diabetes Treatment Satisfaction Questionnaire (DTSQ), comprising 8 items scored on a scale from 0 to 6, at 0, 6, and 18 months. Items 1, 4, 5, 6, 7 and 8 are summed to calculate the Treatment Satisfaction score (range: 0 to 36). The higher the score, the greater the satisfaction with treatment. Item 2 is “perceived frequency of hyperglycemia” and item 3 is “perceived frequency of hypoglycemia.” Both are rated from 0 to 6, and lower scores indicate more ideal glucose levels. [13]

### Dietary intervention

Dietary intervention was performed during the 6-month RCT period. For patients on the CRD, the target total calorie intake was calculated by multiplying the ideal body weight by 28 kcal/kg, according to the guidelines of the Japan Diabetes Society. [14] The total calorie intake consisted of 50–60% carbohydrate, 1.0–1.2 g/kg protein, and the remainder as fat.

For patients on the LCD, the target carbohydrate intake was set as 130 g/day, representing the average minimum requirement stipulated by the Food and Nutrition Board of the Institute of Medicine, as well as the cut-off for a low-carbohydrate diet as defined by Accurso et al. [7, 15, 16] In our RCT, patients were requested to consume equal amounts of carbohydrate at each meal (about 43.3 g). In the Japanese diet, the carbohydrate content in side dishes is about 20 g. [17] Patients were asked to derive about 23.3 g of carbohydrate at each from the staple grain food. Dieticians explained carbohydrate amounts using food choice lists. There were no specific food restrictions apart from the amount of carbohydrates and a recommendation to consume unsaturated fat rather than saturated fat. Patients were basically requested to consume the same amount of carbohydrate 3 times a day. However, for patients who had difficulty doing so, dieticians recommended that they simply limit their overall consumption of carbohydrate to 130 g/day.

The written materials prepared by the study team physicians and dieticians were provided to both groups at the first nutrition meeting, and contained key points on each nutrition therapy in addition to the food choice lists. At the second meeting, a dietician checked the amounts and types of foods consumed based on the 3-day weighed/measured food records prepared by the patients themselves. Every effort was made to explain and rectify any issue that patients found difficult to follow when using the checklists for each nutrition therapy.

Nutritional education sessions at the Outpatient Clinic generally lasted about 30 minutes each. The well-constructed 5-session program over a 6-month period was established specifically for our RCT.

### Statistical analysis

Data were expressed as mean ± SD for normally distributed data, and median (interquartile range) for data with skewed distribution. The Mann-Whitney *U* test and Wilcoxon signed-rank test were used for data analysis. A *p* value <0.05 denoted the presence of a statistically significant difference. All statistical analyses were conducted per protocol using the JMP statistical software package, version 10.0.2 (SAS Institute, Cary, NC).

## Results

**Fig 1:**
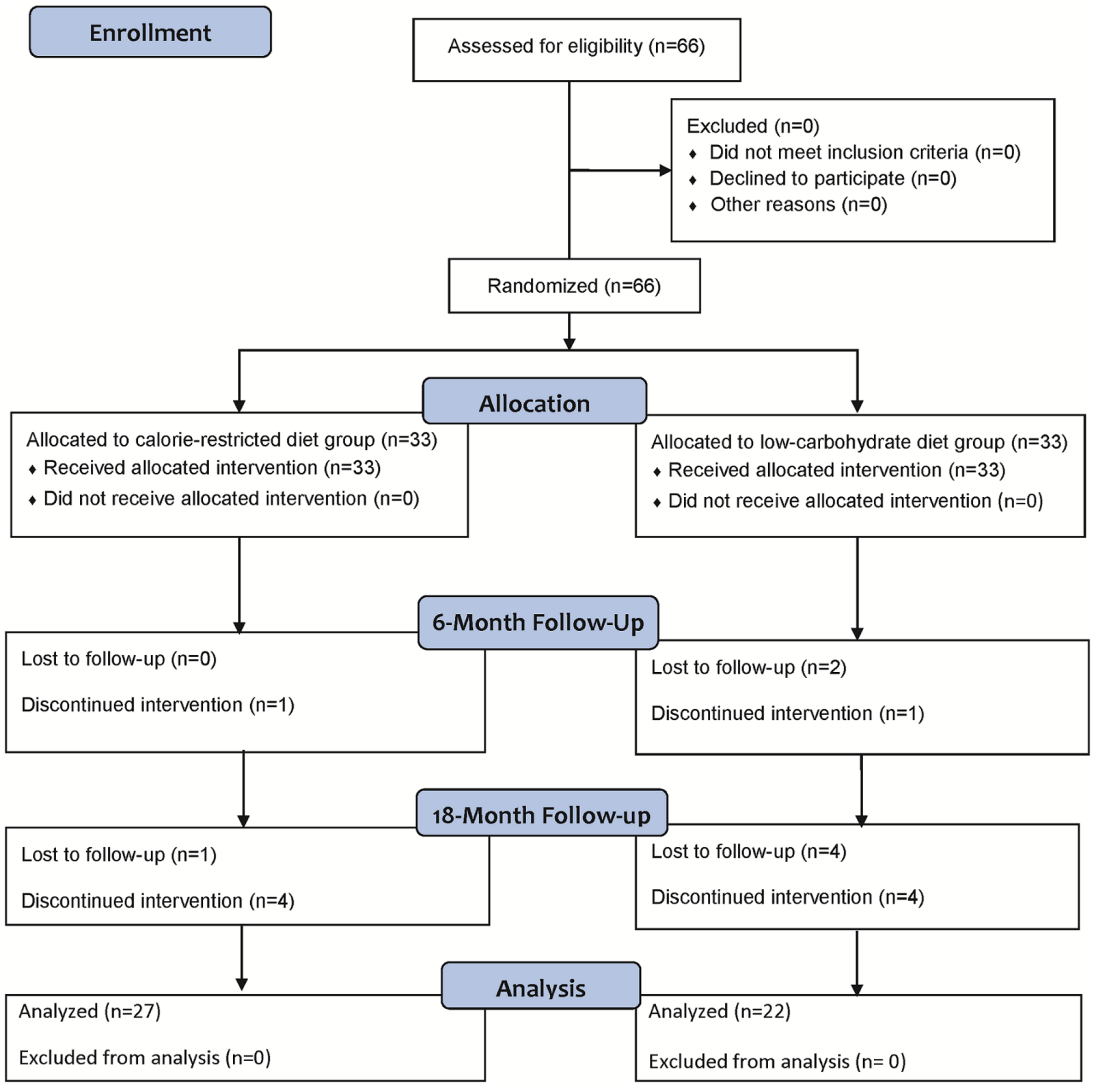
Study flow chart. The baseline data of the study subjects are shown in Tables 1 and 2. Except for age and fat intake, other data were well-matched.

Among 32 patients who finished the RCT in the CRD group, 4 discontinued the intervention (by transferring to the different hospitals) and 1 was lost to follow-up (with admission to the hospital due to the different disease), and thus 27 patients completed the 1-year follow-up. On the other hand, among 30 patients in the LCD group, 4 discontinued the intervention (by transferring to the different hospitals) and 4 were lost to follow-up (with admission to the hospital due to the different disease and diabetes), resulting in 22 patients completing the 1-year follow up (Fig 1). We were therefore able to obtain the body weight and blood data of a total of 49 patients for 18 months, including the 6-month RCT period, in addition to 3-day weighed/measured food records and a completed Diabetes Treatment Satisfaction Questionnaire (DTSQ) from 38 patients (CRD:19, LCD:19) at the end of the follow-up period.

**Table 1:** Characteristics of 49 patients at the RCT baseline, and changes in medication dosages at the end of 1-year follow-up.

	Calorie-restricted diet	Low-carbohydrate diet	P value
**n**	27	22	
**Gender (male/female)**	19/8	16/6	NS (0.86)
**Age (years)**	56.9±9.9	63.0±9.8	0.03
**Height (cm)**	165.9±9.6	163.3±6.2	NS (0.29)
**Body weight (kg)**	73.9 (68.1–88.0)	67.9 (64.8–80.3)	NS (0.52)
**Duration of diabetes (years)**	13.0 (8.0–20.0)	15.5 (8.0–18.5)	NS (0.4)
**Medications for diabetes**			
**Basal supported oral therapy (n)**	1	2	
**Intensive insulin therapy (n)**	10	5	
**Basal insulin (units/day)**	18±7	14±8	NS (0.21)
**Bolus insulin (units/day)**	23±10	15±14	NS (0.15)
**SU/Met/TZD/DPP4i/α-GI/Glinide (n)**	13/19/4/16/8/2	8/16/3/11/7/2	
**GLP-1 receptor agonist (n)**	1	2	
**Medications for other diseases (n)**			
**Antihypertensive agents**	12	9	
**Lipid-lowering agents**	19	15	
**Others**	7	8	
**Changes in medication dosages at the end of the 1-year follow-up period (n)**			
**Increased from baseline**	17	17	
**Reduced from baseline**	11	14	
**Change in insulin dosages (n)**			
**Increased from baseline**	7	4	
**Decreased from baseline**	7	4 (+1 stopped insulin)	

Data are expressed as mean ± SD or median (interquartile range). Met: metformin, TZD: thiazolidine, DPP4i: dipeptidyl peptidase-4 inhibitor, α-GI: alpha-glucosidase inhibitor, NS: not significant.

**Table 2:** Data at baseline, 6 months, and 18 months for 49 patients in the calorie-restricted diet (CRD) group and the low-carbohydrate diet (LCD) group.

Variable	Baseline	6 months	Change from baseline	*P* value (intragroup)	18 months	Change from baseline	*P* value (intragroup)
Body weight (kg)	73.0 (65.6–87.0)	71.1 (64.4–83.9)	-1 (-2.6 to 0.8)	0.03	71.8 (64.0–84.0)	-2 (-3.1 to 0.3)	0.0006
CRD	73.9 (68.1–88.0)	73.9 (66.0–88.2)	-0.6 (-1.2 to 0.8)	NS(0.24)	72.0 (64.0–85.0)	-1.6 (-3.0 to 0.5)	0.01
LCD	67.9 (64.8–80.3)	66.5 (63.7–80.2)	-1.8 (-4.2 to -0.3)	0.03	69.0 (63.5–82.2)	-2.5 (-3.63 to 0.3)	0.01
*P* value (intergroup)	NS (0.33)	NS (0.19)	0.03		NS(0.45)	NS(0.64)	
Body mass index (kg/m^2^)	26.5 (24.8–30.2)	26.2 (23.7–30.1)	-0.36 (-0.89 to 0.29)	0.005	25.9 (23.8–29.7)	-0.67 (-1.13 to 0.12)	0.0001
CRD	26.5 (25.5–30.2)	26.5 (24.7–30.2)	-0.20 (-0.44 to 0.37)	NS(0.18)	25.9 (24.2–30.3)	-0.63 (-1.20 to 0.18)	0.01
LCD	26.1 (24.7–30.0)	25.3 (23.5–29.1)	-0.67 (-1.63 to -0.12)	0.02	26.1 (23.5–29.2)	-0.77 (-1.15 to -0.12)	0.008
*P* value (intergroup)	NS (0.79)	NS (0.27)	0.04		NS(0.77)	NS(0.7)	
HbA1c (%)	8.3 (7.8–9.0)	8.0 (7.0–8.8)	-0.2 (-1.0 to 0.2)	0.008	7.9 (7.1–8.9)	-0.4 (-0.95 to 0.3)	0.007
CRD	8.3 (8.1–9.3)	8.2 (7.7–8.9)	-0.1 (-0.7 to 0.4)	NS(0.29)	8.2 (7.3–9.1)	-0.4 (-0.9 to 0.3)	0.04
LCD	8.0 (7.5–8.9)	7.3 (6.8–8.4)	-0.4 (-1.35 to -0.08)	0.008	7.7 (6.8–8.8)	-0.35 (-1.0 to 0.35)	NS(0.1)
*P* value (intergroup)	NS (0.09)	0.008	NS (0.09)		NS(0.12)	NS(1.0)	
Triglycerides (mg/dL)	143.0 (95.5–229.0)	148.0 (98.0–252.5)	-8.0 (-39.0 to 46.0)	NS(0.61)	135.0 (93.5–193.5)	-6.0 (-48.0 to 40.0)	NS(0.72)
CRD	162.0 (89.0–242.0)	157.0 (116.0–258.0)	-8.0 (-42.0 to 82.0)	NS(0.99)	135.0 (91.0–192.0)	-8.0 (-50.0 to 19.0)	NS(0.29)
LCD	128.5 (101.3–237.5)	134.5 (91.8–205.0)	-10.5 (-36.5 to 30.5)	NS(0.43)	134.0 (107.8–200.3)	9.5 (-43.0 to 55.3)	NS(0.65)
*P* value (intergroup)	NS (0.76)	NS (0.31)	NS (0.62)		NS(0.79)	NS (0.31)	
HDL cholesterol (mg/dL)	47.0 (38.5–56.0)	48.0 (38.0–57.0)	1 (-3 to 4)	NS(0.39)	48.0 (39.5–57.0)	0.0 (-3.0 to 7.0)	NS(0.32)
CRD	47.0 (38.0–55.0)	46.0 (38.0–57.0)	1.0 (-3.0 to 4.0)	NS(0.61)	50.0 (38.0–56.0)	1.0 (-3.0 to 7.0)	NS(0.41)
LCD	44.5 (38.8–60.8)	48.5 (37.0–55.8)	1.0 (-3.0 to 4.3)	NS(0.43)	46.5 (40.0–59.3)	0 (-3.0 to 7.0)	NS(0.6)
*P* value (intergroup)	NS (0.97)	NS (0.91)	NS (0.88)		NS(0.9)	NS(1.0)	
LDL cholesterol (mg/dL)	99.0 (87.0–130.5)	101.0 (85.5–123.5)	0.5 (-18.5 to 8.0)	NS(0.31)	96.0 (77.0–118.0)	-7.0 (-16.8 to 5.3)	0.02
CRD	97.0 (87.8–129.5)	101.0 (89.0–124.0)	3.5 (-9.8 to 8.5)	NS(0.83)	100.5 (73.3–118.0)	-3.0 (-22.0 to 12.5)	NS(0.28)
LCD	103.0 (79.8–134.8)	100.5 (81.5–123.3)	-8.5 (-21.5 to 7.0)	NS(0.17)	93.0 (78.0–117.5)	-8.0 (-16.0 to 3.5)	0.009
*P* value (intergroup)	NS (1.0)	NS (0.63)	NS (0.25)		NS(0.62)	NS(0.57)	
Serum creatinine (mg/dL)	0.69 (0.53–0.81)	0.72 (0.53–0.80)	0.01 (-0.04 to 0.05)	NS(0.62)	0.7 (0.57–0.79)	0.02 (-0.04 to 0.06)	NS(0.46)
CRD	0.66 (0.53–0.76)	0.69 (0.52–0.75)	0.02 (-0.01 to 0.05)	NS(0.2)	0.67 (0.56–0.77)	0.03 (-0.04 to 0.06)	NS(0.19)
LCD	0.76 (0.57–0.93)	0.75 (0.53–0.92)	-0.02 (-0.05 to 0.04)	NS(0.55)	0.75 (0.58–0.90)	-0.01 (-0.06 to 0.04)	NS(0.77)
*P* value (intergroup)	NS (0.18)	NS (0.26)	NS (0.2)		NS(0.2)	NS(0.32)	
Diet							
Energy (kcal/day)	1737 (1457–2006)	1480 (1273–1696)	-237 (-564 to 67)	0.003	1665 (1457–1834)	-123 (-333 to 198)	NS(0.18)
CRD	1668 (1404–1857)	1538 (1424–1810)	41 (-479 to 386)	NS(0.74)	1644 (1481–1756)	20 (-310 to 264)	NS(0.92)
LCD	1793 (1620–2120)	1430 (1164–1598)	-467 (-583 to -108)	0.0001	1732 (1347–1897)	-193 (-635 to 175)	NS(0.07)
*P* value (intergroup)	NS (0.13)	0.04	0.01		NS(0.47)	NS(0.18)	
Protein (g/day)	62 (55–78)	64 (56–73)	-3 (-15 to 8)	NS (0.15)	67 (56–78)	4 (-11 to 11)	NS (0.72)
CRD	61 (53–70)	64 (57–73)	6 (-9 to 14)	NS (0.63)	63 (56–77)	4 (-0.3 to 14)	NS (0.12)
LCD	68 (61–95)	65 (55–74)	-11 (-21 to 1)	0.008	72 (53–80)	4 (-25 to 10)	NS (0.47)
*P* value (intergroup)	NS (0.05)	NS (0.82)	0.04		NS(0.56)	NS(0.38)	
Fat (g/day)	55 (40–70)	52 (41–67)	-5 (-17 to 13)	NS (0.45)	52 (47–61)	-1.9 (-14 to 8.1)	NS (0.38)
CRD	52 (35–62)	49 (37–60)	-6 (-13 to 18)	NS (0.86)	52 (48–61)	-0.02 (-9 to 14)	NS (0.68)
LCD	67 (42–88)	56 (41–69)	-5 (-18 to 8)	NS (0.24)	55 (47–74)	-2 (-17 to 7)	NS (0.07)
*P* value (intergroup)	0.03	NS (0.5)	NS (0.42)		NS(0.73)	NS(0.11)	
Carbohydrate (g/day)	226 (199–252)	178 (143–208)	-51 (-92 to -5)	0.0001	215 (178–249)	-9 (-62 to 16)	NS (0.16)
CRD	211 (191–256)	208 (185–234)	-10 (-64 to 26)	NS(0.33)	215 (189–243)	-6 (-40 to 28)	NS (0.57)
LCD	230 (209–251)	151 (127–170)	-87 (-114 to -44)	0.0001	214 (176–262)	-27 (-62 to 16)	NS (0.13)
*P* value (intergroup)	NS (0.35)	0.0001	0.0003		NS(0.84)	NS(0.6)	
Saturated fatty acids (g/day)	15.3 (9.3–20.4)	14.3 (10.0–18.2)	-0.5 (-4.8 to 4.3)	NS(0.88)	14.2 (11.2–17.3)	-0.2 (-5.0 to 3.3)	NS (0.7)
CRD	13.5 (8.3–17.0)	12.7 (9.1–16.3)	-0.9 (-5.0 to 5.6)	NS(0.91)	14.6 (11.9–16.7)	1.5 (-1.7 to 3.8)	NS (0.37)
LCD	16.2 (11.5–23.4)	16.7 (10.5–21.7)	-0.02 (-4.8 to 3.6)	NS(0.92)	13.5 (10.1–20.1)	-0.4 (-6.1 to 1.2)	NS (0.17)
*P* value (intergroup)	NS (0.07)	NS (0.17)	NS (0.98)		NS(0.77)	NS(0.1)	
Monounsaturated fatty acids (g/day)	21.0 (13.6–26.8)	18.7 (14.5–25.3)	-2.0 (-4.6 to 4.5)	NS(0.65)	19.6 (17.6–22.2)	0.2 (-6.5 to 4.8)	NS(0.69)
CRD	18.1 (13.3–21.7)	18.4 (14.1–22.8)	-0.5 (-4.3 to 5.4)	NS(0.54)	19.9 (15.9–22.0)	0.4 (-4.0 to 8.7)	NS(0.68)
LCD	25.0 (13.9–28.6)	18.9 (14.5–27.6)	-2.3 (-7.5 to 3.9)	NS(0.23)	19.3 (17.7–26.4)	-0.1 (-9.8 to 4.8)	NS(0.24)
*P* value (intergroup)	NS (0.05)	NS (0.68)	NS (0.19)		NS(0.78)	NS(0.29)	
Polyunsaturated fatty acids (g/day)	11.3 (8.5–14.9)	10.3 (9.0–13.5)	0.1 (-4.3 to 1.8)	NS (0.55)	10.6 (9.2–13.0)	1.0 (-4.8 to 1.8)	NS(0.73)
CRD	10.2 (7.4–12.5)	10.0 (8.6–13.4)	1.3 (-2.1 to 2.5)	NS (0.65)	10.3 (9.3–12.2)	1.2 (-2.7 to 2.7)	NS(0.48)
LCD	13.3 (10.5–16.6)	10.3 (9.7–13.8)	-0.6 (-5.4 to 1.0)	NS (0.2)	11.4 (9.1–13.1)	-0.9 (-6.5 to 1.8)	NS(0.28)
*P* value (intergroup)	NS (0.05)	NS (0.44)	NS (0.22)		NS(0.23)	NS(0.22)	
Perception of hyperglycemia (points)	4.0 (3.0–5.0)	3.0 (2.0–4.3)	-1 (-2 to 0.3)	0.02	3.0 (2.0–4.0)	-1 (-2 to 0.0)	0.009
CRD	4.0 (3.0–5.0)	4.0 (2.0–4.0)	0.0 (-2.0 to 0.0)	NS (0.19)	3.0 (2.0–4.0)	-1.0 (-2.0 to 0.0)	NS (0.05)
LCD	4.0 (3.0–5.0)	3.0 (1.0–5.0)	-1.0 (-2.0 to 1.0)	NS (0.07)	3.0 (2.0–4.0)	-1.0 (-2.0 to 0.0)	NS (0.11)
*P* value (intergroup)	NS (0.9)	NS (0.38)	NS (0.56)		NS(1.0)	NS(0.95)	
Perception of hypoglycemia (points)	0.0 (0.0–1.0)	1.0 (0.0–2.0)	0.0 (0.0–1.0)	NS(0.28)	1.0 (0.0–2.0)	0.0 (0.0–1.0)	0.046
CRD	0.0 (0.0–2.0)	1.0 (0.0–3.0)	0.0 (0.0–0.0)	NS (0.38)	1.0 (0.0–2.0)	0.0 (0.0–1.0)	NS (0.44)
LCD	0.0 (0.0–1.0)	1.0 (0.0–2.0)	0.0 (0.0–1.0)	NS (0.39)	1.0 (0.0–2.0)	0.0 (0.0–1.0)	NS (0.06)
*P* value (intergroup)	NS (0.58)	NS (0.36)	NS (0.82)		NS(0.83)	NS(0.41)	
DTSQ satisfaction score (points)	21.0 (18.8–26.3)	23.0 (19.8–26.0)	0.5 (-2.3 to 5.3)	NS(0.36)	23.0 (19.0–28.0)	1 (-3.3 to 5.3)	NS(0.3)
CRD	22.0 (18.0–26.0)	23.0 (19.0–25.0)	-1.0 (-4.0 to 5.0)	NS(0.79)	21.0 (19.0–28.0)	0 (-4.0 to 5.0)	NS(0.84)
LCD	21.0 (19.0–27.0)	23.0 (21.0–27.0)	1.0 (-2.0 to 6.0)	NS(0.28)	24.0 (20.0–28.0)	1.0 (-1.0 to 6.0)	NS(0.17)
*P* value (intergroup)	NS (0.76)	NS (0.4)	NS (0.51)		NS(0.51)	NS(0.4)	

Data are expressed as median (interquartile range). The data of nutrition and DTSQ questionnaires were from 38 patients who submitted 3-day weighed/measured food records and DTSQ questionnaires at the end of the 1-year follow-up period. DTSQ: diabetes treatment satisfaction questionnaire. "Change from baseline" is shown as the change in actual value between baseline and 6 months / 18 months.

Five patients in the CRD group and 4 in the LCD group increased the dosage of their oral medications (CRD: metformin, 4; sulfonylurea, 1; LCD: metformin, 3; thiazolidine, 1). Five patients in the CRD group and 6 in the LCD group decreased their oral medication dosages (CRD: metformin, 2; Dipeptidyl peptidase-4 inhibitors [DPP4-I], 1; sulfonylurea, 1; thiazolidine, 1; LCD: metformin, 2; alpha-glucosidase inhibitor [α-GI]: 1; sulfonylurea, 3). Eleven patients in both the CRD and LCD groups started new oral medications (CRD: metformin, 2; DPP4-I, 2; Sodium-glucose co-transporter-2 inhibitors [SGLT2-I], 6; thiazolidine, 1; LCD: metformin, 1; DPP4-I, 2; glinide, 3; SGLT2-I, 4; sulfonylurea, 1). Six patients in the CRD group and 8 in the LCD group discontinued oral medications (CRD: metformin, 1; α-GI, 1; DPP4-I, 1; sulfonylurea, 1; thiazolidine, 2; LCD: DPP4-I, 1; sulfonylurea, 5; α-GI, 2). One patient in the CRD group and 2 in the LCD group began treatment with a GLP1 receptor agonist. The basal insulin dose was increased in 4 patients in the CRD group and 3 patients in the LCD group, and decreased in 3 patients in the CRD group and 1 patients in the LCD group. One patient in the LCD group discontinued basal insulin treatment. The overall change in the basal insulin dose was +2 (-3 to +5) units in the CRD group and +2 (-9 to +3) units in the LCD group. The bolus insulin dosage was increased in 3 patients in the CRD group and 1 patient in the LCD group, and decreased in 4 patients in the CRD group and 3 patients in the LCD group. The overall change in the bolus insulin dose was -1 (-2 to +2) units in the CRD group and -5 (-7 to +4) units in the LCD group.

Consistent with the findings of previous studies, [2–8] patients in the LCD group, compared with those in the CRD group, showed a significant decrease from baseline to the end of the 6-month RCT period in terms of calorie intake, carbohydrate intake, body weight, BMI, and HbA1c in our previous study with 62 patients. [1] However, no differences in these factors were observed between the 2 groups at 18 months after baseline (Table 2). Also, changes in other clinical data, including TG, HDL-C, LDL-C, and serum creatinine were not significantly different between the 2 groups.

Combining the data of both groups, a significant decrease from baseline to 18 months was observed in body weight (-2 [-3.1 to 0.3] kg, p<0.01), BMI (-0.67 [-1.13 to 0.12] kg/m^2^, p<0.01), and HbA1c (-0.4 [-0.95 to 0.3]%, p<0.01), even though there were no significant changes in energy intake (-123 [-333 to 198] kcal/day) or carbohydrate intake (-9 [-62 to 16] g/day). In addition, a significant improvement in LDL-C (-7.0 [-16.8 to 5.3] mg/dl, p<0.05) was observed from baseline to the study endpoint at 18 months (Table 2).

During the RCT, confirmed and non-confirmed hypoglycemia were observed in 3 and 1 patients, respectively, in the LCD group. After the RCT, there were no cases of hypoglycemia in the LCD group. In the CRD group, there were no cases of hypoglycemia during the entire 18-month study period. No other important harms or unintended effects in each group were observed.

Originally, we planned to analyze the data without the adjustment for variables. However, it is also worth to analyze with adjustment for key variables. Thus, follow-up data (month 6 and 18) were analyzed again using a mixed-effects model for repeated measures with a model including treatment group and time (month), interactions between treatment group and time, and age, duration of T2DM, baseline values of each analyzed variable, HbA1c, BMI, energy and carbohydrate as fixed effects; an unstructured covariance was used to the covariance within-subject variability. The results of the adjusted analysis were almost same with that of the original analysis, and the conclusion of the study did not change.

## Discussion

This study was an extension of our 6-month intervention study in which we found that the LCD was superior to the CRD in terms of HbA1c and BMI. One year after the end of the RCT, we found no significant differences between the CRD and LCD groups in either ΔHbA1c or ΔBMI from baseline. However, combining the data of both groups, a significant decrease in HbA1c and BMI from baseline was observed at 18 months after the beginning of the study.

The carbohydrate intake at the end of the RCT was about 149 g/day in the LCD group compared to about 198 g/day in the CRD group. [1] Even though the goal of 130 g/day of carbohydrate was not achieved, the patients in the LCD group demonstrated rapid improvement in HbA1c and BMI during the 6-month RCT. This could be mainly due to significant reduction in energy intake. We do not know the exact reason of success in significant reduction in energy intake in LCD group. However, we guess that the patients in LCD intervention group payed attention to “the carbohydrate amount” in daily life. This change of attitude could contribute to success in the decrease of the energy intake.

One year after the RCT, HbA1c and BMI were comparable between the groups, probably because patients were unable to maintain a low carbohydrate intake in the absence of the systematic intervention provided during the RCT, or just did not like following the strict diet. Indeed, at the end of the follow-up period, the carbohydrate intake was 215 (189 to 243) g/day in the CRD group and 214 (176 to 262) g/day in the LCD group. This result suggests the difficulty of maintaining a reduced carbohydrate intake in Japanese patients with type 2 diabetes mellitus. Previous studies showed that carbohydrate intake was higher in Japan than in Western countries. [18–20] The average calorie intake of Japanese was 1863 kcal/day in 2014, of which 57.3% was derived from carbohydrates. [20] On the other hand, the average calorie intake of Americans was 2137 kcal/day, of which 49.2% was from carbohydrates. [19] Moreover, the major sources of carbohydrates for Japanese are rice and processed rice (47.0%), which together are traditionally regarded as the staple food. [20] However, in the US, the primary carbohydrate sources are soft drinks and soda (13.9%), yeast breads and rolls (10%), and cakes, cookies, quick bread, pastry, and pie (8.3%), while rice and cooked grains account for only 3.1% of the total carbohydrate intake. [18, 21] Accordingly, it might be more difficult for Japanese than Americans to maintain a reduced carbohydrate intake.

On the other hand, for individuals in all countries it is difficult to sustain nutritional change if the assigned diet differs markedly from the usual diet. A systematic review of the effect of macronutrients on glycemic control pointed out the insufficiency of dietary adherence. [22] In addition, in Iqbal’s 24-month study, the subjects were prescribed a diet with <30 g of carbohydrates/day; however, data from 3-day food diaries revealed a mean carbohydrate intake of 192.8 g/day at 2 years after the end of the intervention. [22, 23]

Although no significant differences between the CRD and LCD groups were observed for either ΔHbA1c or ΔBMI from baseline, combined data of the 2 groups showed a significant decrease of HbA1c and BMI at 1 year after the end of the RCT. The systemic nutrition education provided during the RCT period might explain the long-lasting improvement in both groups. The nutrition education in this study was presented in the RCT by skilled dieticians during 5 individualized sessions at 0, 1, 2, 4, and 6 months. The American Diabetes Association (ADA) recommends individualized medical nutrition therapy (MNT) by a registered dietician who is knowledgeable and skilled in providing diabetes-specific MNT. [24] The ADA also found that MNT delivered by a registered dietician was associated with HbA1c decreases of 0.5–2% in patients with T2DM. [24–28]

MNT provided to patients in the LCD group rapidly improved HbA1c and BMI, and the improvement was still present at 1 year after the RCT. MNT for patients in the CRD group did not result in rapid improvement, but there was still gradual progress; at the end of the follow-up period, the change in HbA1c and BMI from baseline was almost same as that in the LCD group.

At the end of the study, the overall population demonstrated no significant change in energy intake (-123 ([333 to 198] kcal/day) or carbohydrate intake (-9 [-62 to 16] g/day) relative to baseline, mainly because the energy intake in the CRD group did not decrease. It was difficult to precisely evaluate nutritional data since only 38 of 49 patients completed the 1-year follow-up. However, regarding the percentages of total calories, those from fat, carbohydrate, and saturated fatty acids tended to decrease, while those from monounsaturated and polyunsaturated fatty acids tended to increase. These changes may have contributed to the improvement in HbA1c and BMI. Indeed, some randomized controlled trials, including those in patients with type 2 diabetes mellitus, have reported that a Mediterranean diet rich in monounsaturated fats can improve glycemic control and blood lipids. [24, 29–33]

Our study has several limitations. First, as mentioned in the previous RCT, the number of patients was limited, and they could not be blinded due to the characteristics of the dietary therapy. Second, at the end of follow-up, only 38 of 49 patients provided records of their nutritional data and thus only these patients were analyzed. Third, the fact that patients self-reported their 3-day weighed/measured food records for evaluation of dietary intake and their use of multiple medications could have biased the study results. Fourth, the clinicians tried to adjust patients’ medications, especially after the RCT. However, there were no obvious imbalances in medication adjustment between the 2 groups.

In conclusion, we demonstrated that a LCD with 130 g/day of carbohydrate intake for 6 months was more effective than a CRD in Japanese patients with T2DM who could not achieve good glycemic control. However, after the RCT, in the absence of the intervention, patients in the LCD group did not maintain their low carbohydrate intake. Nonetheless, these patients showed a persistent improvement in HbA1c and BMI for 1 year after the RCT. Further, the patients in the CRD group demonstrated a gradual improvement in HbA1c and BMI during the 1 year after the RCT. At the end of the follow-up period, both groups showed a significant improvement relative to baseline. The CRD and LCD were both well-constructed and individualized nutrition therapy programs, and were found to effectively reduce HbA1c and BMI for an extended period beyond the end of the interventions. Further research is needed to find interventions to sustain the results at 6 months, including more frequent dietitian contact.

## Supporting information

S1 Text Study protocol. (DOCX)

S2 Text Study protocol Japanese. (DOCX)

S3 Text CONSORT 2010 checklist. (DOC)
