# Supplementary material for: One year follow-up after a randomized controlled trial of a 130 g/day low-carbohydrate diet in patients with type 2 diabetes mellitus and poor glycemic control
Source: PLoS One. 2017 Dec 4;12(12):e0188892. doi: 10.1371/journal.pone.0188892 (PMC5714344; doi:10.1371/journal.pone.0188892)
Supplement: S1 Text — (DOCX) [file pone.0188892.s001.docx]

**Study Protocol** (Translation)

**A randomized controlled trial of 130 g/day low-carbohydrate diet in type 2 diabetes with poor glycemic control**

**Submitted on Feb. 26 / 2013, Revised on April 20 / 2013**

**Purpose, Background**

Diet is one of the basic therapies for patients with type 2 diabetes mellitus (T2DM). Both calorie-restricted diets (CRDs) and low-carbohydrate diets (LCDs) are regarded as useful therapies for patients in Western countries. However, differences in food cultures among countries markedly affect the efficacy of diet therapies, thus it is important to evaluate the methodology of each diet therapy in individual countries. Regarding Japanese patients, except for 1 small randomized controlled trial (RCT), no previous RCTs have compared the effects of LCDs and CRDs. Thus, we conduct a 6-month RCT to compare the outcome of a LCD with that of a CRD, limiting carbohydrate intake to 130 g/day presented by R Barnstein^1)^, in obese Japanese patients with uncontrolled T2DM.

1 Richard Barnstein, “Review: Dietary carbohydrate restriction in T2DM and metabolic syndrome: time for a critical appraisal” (Nutrition and Metabolism 2008)

**Design**

Prospective, two-arm, randomized controlled study.

**Participant**

We recruit the patients with type 2 diabetes mellitus whose HbA1c was more than 7.5 % and BMI is more than 23 kg/m^2^ in spite of the repeated history (more than twice) of receiving the guidance of CRD by qualified dietician from the outpatient clinic of Juntendo University Hospital. The patients who fulfilled the following criteria at registration are included in the study: 1) 75 years > age > 20 years, 2) HbA1c (NGSP) was more than 7.5% for more than 3 months, and HbA1c fluctuations are within ± 0.5%. However, the selected type 2 diabetes patients are excluded from the study when any of the following conditions are diagnosed at registration: 1) proliferative retinopathy, 2) severe neuropathy, 3) serious kidney disease (serum creatinine level > 2.0 mg/dL and/or with microalbuminuria), 4) serious liver disease except fatty liver (aspartate aminotransferase and/or alanine aminotransferase levels >100 IU/L), 5) acute heart failure within 3 months or apparent chronic heart failure, 6) active malignancy, 7) serious pancreatic disease, 8) pregnancy, 9) serious infectious disease, 10) trauma injury, 11) heavy drinker of alcohol and 12) not suitable for the study.

**Recruitment**

Recruitment of 66 patients (33 patients in each group) starts from September in 2013.

Intervention period: 6 months.

Follow-up period: One year after the end of the intervention.

Intervention is held at Juntendo University Hospital.

**Study design**

This open-label, two-arm, randomized controlled study is performed at the outpatient clinic of Juntendo University Hospital. 66 patients meeting the criteria are assigned randomly to either LCD or CRD for 6 months.

**Intervention**

The study is performed for 6 month, and the patients are followed up by the same medical doctors of the outpatient clinic and qualified dieticians at 0, 1, 2, 4, 6 month of the period. At the beginning, the study team doctors explain to the patients directly about the protocol. At all visits, the changes of medication are made by their outpatient doctors whenever it is necessary.

At all visits, the body weight is measured by the dietician at the nutrition counseling room and is used to calculate BMI. Blood samples are obtained after overnight fast. Serum lipids (total cholesterol [T-CHO], HDL-cholesterol [HDL-C], LDL-cholesterol [LDL-C], Triglyceride [TG]), fasting blood glucose, HbA1c, liver enzymes, serum creatinine and microalbuminuria are measured with standard techniques at 0, 1, 2, 4, 6 month. At the beginning and the end of the study, the patients complete the Diabetes Treatment Satisfaction Questionnaire (DTSQ).

After the end of the 6-month RCT, the patients are allowed to manage their own diets and make periodic visits to the outpatient clinic of Juntendo University Hospital. We obtain their clinical and nutrition data 1 year after the end of the RCT.

**Dietary Intervention**

For patients on CRD, the target of total calorie intake is calculated by multiplication of the ideal body weight and 28kcal/kg, following the guideline of the Japan Diabetes Society. The percentage of carbohydrate in the total calorie is 50 ~ 60 %, and the protein is 1.0 ~ 1.2g/kg. The rest of the calorie is covered with the fat.

For patients on LCD, the target carbohydrate intake is decided to be 130g per day. In this study, the patients are requested to take the equal amount of carbohydrate at each meal (about 43.3g each). The carbohydrate in side dishes is about 20g each in Japanese diet. The patients are requested to take carbohydrate about 23.3g each time from the staple food. The dieticians emphasized to explain how much the patients can eat carbohydrate by using the food choice lists. The patients are basically requested to take the same amount of carbohydrate three times a day, but if it is impossible for some patients, the dieticians explain to take 130g carbohydrate per day.

The written materials made by the study team doctors and dieticians are supplied to the both groups containing the key points of each diet therapy with food choice lists at the first nutrition meeting. From the second time, using the three days weighed / measured food records brought by the patients themselves, the dietician checks the amount and the type of foods precisely. If they find the points that the patients can not follow by using the check lists of each diet therapy, they guided that point specifically.

**Endpoint**

The primary endpoint is a change in HbA1c level from baseline to the end of the study. The secondary endpoint is changes in BMI and lipid metabolism from baseline to the end of the study and frequency of hypoglycemia during the study period.

**Informed consent**

Written informed consent is obtained from each patient before enrollment in the study.

**Privacy**

Privacy of the patients is protected.

**Study cancellation**

The study will be cancelled

1 ) when the patients offer the cancellation.

2) when it is difficult to continue the study with the severe adverse effects.

3) when there is severe protocol violation.

4) when the team doctors judged the necessity of study cancellation.

**Study team**

Juntendo Universit, Graduate School of Medicine

Department of Metabolism and Endocrinology

Akio Kanazawa, Fuki Ikeda, Junko Sato

Phone:03-5802-1579

Juntendo University Hospital

Department of Nutrition

Sumiko Makita
