# Supplementary material for: One year follow-up after a randomized controlled trial of a 130 g/day low-carbohydrate diet in patients with type 2 diabetes mellitus and poor glycemic control
Source: PLoS One. 2017 Dec 4;12(12):e0188892. doi: 10.1371/journal.pone.0188892 (PMC5714344; doi:10.1371/journal.pone.0188892)
Supplement: S2 Text — (DOCX) [file pone.0188892.s002.docx]

**2型糖尿病患者における糖質制限食の有効性と安全性**

調査実施計画書

■作成日・承認日・改定日

　2013年　2月　26日作成

　2013年　4月　20日改定

**[目的・背景］**

食事療法は糖尿病患者にとっての重要な治療法のひとつである。しかし現在の糖尿病食はエネルギー制限食であり、満足感が得られないとして守れない2型糖尿病患者も多い。そうしたなか糖質制限食が注目されているが、1日の糖質を50g以下にするアトキンスダイエットなどの過激な糖質制限を行っている人々もいるため、その安全性や糖尿病患者にとっての有効性を正確に評価する必要がある。

アメリカの医師バーンスタインは糖質制限食の炭水化物摂取量を1日130g以下としており^1^、アメリカ糖尿病学会などもこの定義を参考にしている。糖質制限食に対して否定的であったアメリカ糖尿病学会も2008年には肯定的見解を出し、その有益性保証期間を2011年に1年間から2年間に延長している。

糖質制限食はすべての2型糖尿病患者に適応できるわけではない。小児や妊婦、また蛋白制限食が必要な腎症の患者らは除外する必要がある。またインスリン注射やSU薬を使用している患者も、きめこまかな調整を行わないと重篤な低血糖を起こす可能性がある。

今回、順天堂大学医学部附属順天堂医院糖尿病内分泌内科に通院するエネルギー制限食を実践できない2型糖尿病患者を対象として、栄養部の協力のもと糖質制限食の有効性と安全性を検証する。

1 Richard Barnstein, “Review: Dietary carbohydrate restriction in T2DM and metabolic syndrome: time for a critical appraisal” (Nutrition and Metabolism 2008)

**［試験デザイン］**

Prospective, two-arm, randomized controlled study

**［対象］**

20歳から75歳までのエネルギー制限食が効果的に実践できない2型糖尿病患66人。

エネルギー制限食である通常の栄養指導を2回行い、3-4か月経過してもHbA1cが0.5％以上改善しない人。

現行の治療法は問わないが、インスリンやSU薬使用者らは低血糖の危険を考慮し外来医の判断において薬量を十分に減らすことを前提条件に登録可能。

**選択基準**

1. 試験期間中のいかなる薬剤の中止、追加 (インスリンの追加、増量を含む)も可とす

る。低血糖などの有害事象を避けるための変更も医師の判断により可能とする。

1. 年齢は満20歳以上とする(同意取得日の年齢を基準とする)。
2. 性別は問わない。
3. 本試験への参加について同意の能力を有し、同意文書およびその他の説明文書を

読め、かつ理解できる患者。

**除外基準**

次のいずれかに該当する患者は対象から除外する。

1. HbA1c7.4%以下の患者。

2. BMI22以下の患者。

3.　 登録前の糖質が130g/日よりも少ない患者。

登録前の摂取エネルギー量が規定量（IBW×27キロカロリー）よりも少ない患者。

4. 肝硬変などの重篤な肝疾患のために血糖コントロールが困難な患者。脂肪肝は除く。

5. 試験開始前のeGFRが60未満の患者。血清クレアチニン値が2.0mg/dL以上の患者。尿定性で1+以上の尿蛋白が検出される患者。

6. 顕在性の心不全症例および試験開始前3ヶ月以内 に心筋梗塞を起こした患者。

7. 急性膵炎、慢性膵炎などの重篤な膵疾患のために血糖コントロールが困難な患者。

8. 担癌患者。治癒している場合は除く。

9. 高度な糖尿病性神経障害を有する患者。

10. 増殖性網膜症を有する患者。

11. 重篤な感染症、重篤な外傷のある患者。手術前後である患者。

12. 妊婦または妊娠している可能性のある女性、妊娠を希望している女性および授乳中

の女性。

13. その他、試験担当医師が不適当と判断した患者。

**［目標症例数、調査期間、調査施設］**

目標症例数：計66名（各群33名）リクルート期間2013年9月～

調査期間：介入期間は6カ月

終了1年目の通常採血データ追跡

調査施設：順天堂大学医学部附属順天堂医院

**［方法］**

（1）調査スケジュール

・対象患者に対し通常栄養指導を2回施行し、糖尿病の改善効果のない場合は本スタデ

ィに登録。ランダムに以下の2群に振り分ける。

1：通常の糖尿病栄養指導継続。

2：糖質制限食に切り替える。

　・栄養指導は0, 1, 2 ヶ月目までは毎月施行。登録時の栄養指導で除外基準にあてはまる食事をしていることが判明した場合は除外とする。その後外来診療にあわせて2ヶ月ごと（4, 6ヶ月目）に施行。また毎回の栄養指導時に直近3日間で食べたものを記載の上持参してもらう。

栄養指導時は栄養科作成の「患者の理解度の確認事項」をもとに経過観察を行う。

・食事内容の推移に関して客観的なデータとしてBDHQ (Brief-type diet history questionnaire)を施行。また心理学的な問題のフォローとしてDTSQ (Diabetes

Treatment Satisfaction Questionnaire) を施行する。いずれも0, 6 ヶ月目での施行。

・介入は6カ月のみだが、その後の通常採血データも終了1年目まで追跡。

1年目には食事内容の確認のためにBDHQを施行。

（2）糖質制限食の指導ポイント

・一日の糖質は100g/日～130g/日とする。

・栄養科作成の「糖質組み合わせシート」を使用する。このシートでは一日三回の

　食事時に主食として糖質約20g＋副食・その他の糖質として約20g摂取することを

　目標とするが、今回は通常の栄養指導が守れない2型糖尿病患者が対象であり、

イモ類と果物以外の副食は基本的には自由に食べていいこととする。

菓子、アルコール類は血糖をあげないように工夫したものなら可とする。

・3ヶ月経過後に「糖質組み合わせシート」での継続が難しい症例では、一日摂取糖質量を守ることができれば、夕食分の主食の糖質を他の時間に摂取するなど、摂取時間の変更は可能とする。たとえば夕食時は糖質フリーの飲料と副食のみとし、朝昼に主食分の糖質を増やす、なども継続的でなければ認めることとする。

・同様に3ヶ月経過後に継続が難しい症例では、ご飯のかさを増すための工夫として

　マンナンヒカリなどの補助食品や、糖質制限菓子などの紹介も行うが、患者が使用する場合にはその頻度や量などを聴取する。

・栄養指導に参加しない、食事記録を持参しないことが3回続いた場合はドロップアウトとみなすが、以後データフォローは行う。

（3）観察および検査項目

　　1）患者背景

　性別、年齢、糖尿病発症年齢、合併症、身長、体重

2）治療内容 およびコントロール状況、低血糖頻度等

3）食事内容、遵守率　（BDHQおよび栄養科の「患者の理解度の確認事項」を使用）

4）研究開始時および終了時に採血、採尿を行う。

一般血液検査:　赤血球数、白血球数、ヘモグロビン、ヘマトクリット、

血小板数
血液生化学検査: T-Chol、TG、HDL-C、AST(GOT)、ALT(GPT)、γ-GTP、BUN、クレアチニン、尿酸、空腹時血糖、血中ケトン体、 Na、K、 Cl、 HbA1c、尿定性、尿沈渣、尿中Alb／Cr、尿中ケトン体

　　5）心理学的評価　（DTSQ使用）

**［主要評価項目］**

　　HbA1cの改善

**[副次評価項目]**

1) 体重変化

2) 低血糖頻度

3) 摂取糖質量の遵守率

4) 脂質代謝 (総コレステロール、LDLコレステロール、HDLコレステロール、中性脂

　 肪の変化）

**［インフォームドコンセント］**

　　全ての被験者から、文書にてインフォームドコンセントを得る。この試験への参加は、患者の自由意思によって決めることができる。試験への参加を拒否することも可能である。患者は、この研究への参加を断っても、今後の治療内容を含めて、なんら不利益をこうむることはない。またこの試験への参加に一度同意した場合でも、患者の自由意思によりいつでもそれを撤回できる。

**［プライバシー］**

被験者に関する全ての個人的データは、順天堂大学医学部糖尿病内分泌内科にて一括管理し、個人情報保護法遵守のもと、厳密に保護する。この研究を通じて得られたデータは、担当医師と研究の管理者、データ管理者以外の目にふれることなく、患者の氏名や個人を特定できるような情報が研究結果の報告や発表に使用されることはない。検査結果は医師の守秘義務に基づき、患者自身以外には決して知られることはない。今回の結果を集計し、学会や医学雑誌などに発表するが、この場合も患者の名前や個人に関する情報を一切明らかにしない。

**［試験の中止基準］**

1. 被験者が試験の中止を申し出た場合。
2. 有害事象の発現により，試験の継続が困難と判断された場合。
3. 重大なプロトコール違反があった場合。
4. その他、試験担当医師が試験を中止する必要があると判断した場合。

**［研究結果の発表と個人情報の保護］**

すべての検討が終了次第、学会発表および、医学論文として発表する予定であるが、その際、個人の情報や記入された内容が外部にもれることは一切ない。

**[健康被害等に対する補償]**

臨床試験に参加している期間中または終了後に、予測できなかった健康被害が生じた場合は通常の診療における健康被害に対する治療と同様、保険診療として適切な対応を行う。よって治療費の自己負担分に関しては患者の自己負担とする。

この臨床試験では、お見舞金や各種手当など健康被害に対して、特別に経済的な補償は準備していない。なお、医薬品副作用被害救済制度の対象となる場合には、診断書等の書類を作成する。

**［試験責任者］**

順天堂大学医学部 糖尿病内分泌内科

試験責任医師：　糖尿病内分泌内科、准教授　金澤　昭雄

試験分担医師：　糖尿病内分泌内科、教授　綿田　裕孝

　糖尿病内分泌内科、准教授　池田　富貴

糖尿病内分泌内科、大学院生　佐藤　淳子 (データ管理者)

緊急連絡先：03-5802-1579 　医局：佐藤　淳子

研究協力者：順天堂大学医学部附属順天堂医院栄養科

栄養士: 栄養科、主任　牧田寿美子
